# Colloidal Quantum Dot Nanolithography: Direct Patterning via Electron Beam Lithography

**DOI:** 10.3390/nano13142111

**Published:** 2023-07-20

**Authors:** Taewoo Ko, Samir Kumar, Sanghoon Shin, Dongmin Seo, Sungkyu Seo

**Affiliations:** 1 Department of Electronics and Information Engineering, Korea University, Sejong 30019, Republic of Korea; rdks95@korea.ac.kr (T.K.); skumar@korea.ac.kr (S.K.); ghost10s@korea.ac.kr (S.S.); 2 Department of Electrical Engineering, Semyung University, Jecheon 27136, Republic of Korea

**Keywords:** quantum dots, nanolithography, patterning methods, electron beam lithography, colloidal quantum dots

## Abstract

Micro/nano patterns based on quantum dots (QDs) are of great interest for applications ranging from electronics to photonics to sensing devices for biomedical purposes. Several patterning methods have been developed, but all lack the precision and reproducibility required to fabricate precise, complex patterns of less than one micrometer in size, or require specialized crosslinking ligands, limiting their application. In this study, we present a novel approach to directly pattern QD nanopatterns by electron beam lithography using commercially available colloidal QDs without additional modifications. We have successfully generated reliable dot and line QD patterns with dimensions as small as 140 nm. In addition, we have shown that using a 10 nm SiO_2_ spacer layer on a 50 nm Au layer substrate can double the fluorescence intensity compared to QDs on the Au layer without SiO_2_. This method takes advantage of traditional nanolithography without the need for a resist layer.

## 1. Introduction

Colloidal quantum dots (QDs) are semiconducting crystal structures with sizes between 2 and 20 nm, and their energy band gap can be tuned depending on their size [1]. These QDs exhibit remarkable properties, including tunable emission wavelengths, high fluorescence efficiency, exceptional photostability, and excellent optical properties, which make them suitable for various applications [2,3,4,5,6]. Patterning of QDs is a rapidly growing research area. Numerous studies focus on the use of patterned QDs for various applications such as nanoelectronics, complex optical structures, and metamaterials [7,8,9]. To improve the resolution and performance of QD patterns, researchers have explored new materials, patterning techniques, and changes in processing parameters [10,11,12]. Several patterning methods have been developed, including inkjet printing [13], transfer printing [14,15], and dip-pen nanolithography [16]. However, these methods often lack the precision and reproducibility necessary to produce accurate and intricate submicron patterns, which are critical for advanced technologies.

Photolithography has also been used to pattern colloidal QDs by light-driven ligand crosslinking [7,17]. For example, Wang et al. developed inorganic ligand molecules attached to the surface of QDs that can transform upon irradiation with light of different wavelengths (254–450 nm) and even electron beams (e-beams) [18,19]. However, these methods require the use of custom crosslinking ligands, which limits their wide application and often requires the use of photoresist to define patterns, increasing the cost and adding an additional step to the process [14].

To overcome the limitations associated with photolithography, direct patterning of sample materials using electron beam lithography (EBL) has gained importance. EBL offers advantages such as higher speed compared to conventional lithography processes that require deposition, etching, and photoresist removal, as well as higher resolution capabilities that enable the fabrication of structures with a precision of 100 nm or better [20]. However, in previous studies, QDs were usually chemically functionalized with custom-made ligands prior to EBL patterning.

In this study, we present a novel approach to produce QD nanopatterns using EBL without further modification of commercially available colloidal QDs. First, we determined the optimal e-beam concentration and dose for QD nanostructure fabrication. We then fabricated QD line and dot patterns with a minimum width of 140 nm. We also investigated the use of surface plasmons (SP) to increase the fluorescence intensity of the patterns by fabricating the QD nanopatterns on multilayers of Cr, Au, and SiO_2_. The thickness of the Au layer was optimized to achieve maximum QD fluorescence efficiency, resulting in an impressive enhancement of up to 170%. This research demonstrates the feasibility and efficacy of direct QD nanopatterning with EBL using commercially available colloidal QDs and highlights the potential of SP-based strategies to enhance fluorescence intensity. By eliminating the need for specialized ligands and taking advantage of EBL, our approach offers a promising pathway for precise QD nanopatterning, laying the foundation for advanced nanoscale devices and applications.

## 2. Materials and Methods

**Figure 1:**
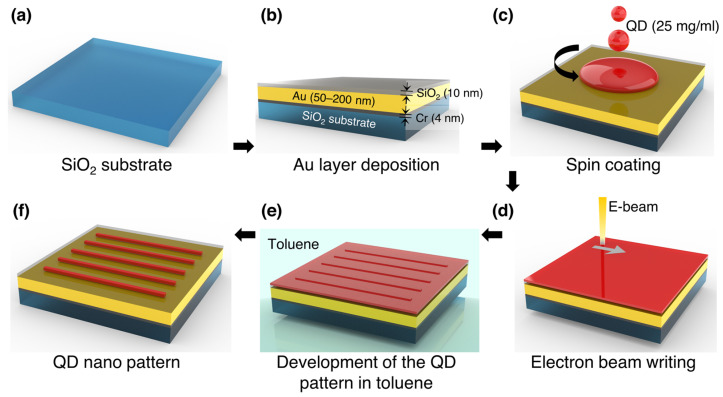
Schematic diagram showing the main steps in the fabrication of quantum dot (QD) patterns using e-beam lithography.

CdSe/ZnS QDs (CZO-530T, CZP-620T) surface-capped with oleic acid were purchased from ZEUS Co., Ltd. (Osan, Republic of Korea). The fabrication process of QD nanopatterns by EBL is shown in Figure 1.

Glass slides (10 × 10 mm^2^) were sonicated, washed sequentially with acetone, isopropanol, and deionized water for 5 min, and then dried with N_2_ gas for 1 min (Figure 1a). Using e-beam physical vapor deposition (EB-PVD) (Daon-1000E; DAON Co. Ltd., Daejeon, Republic of Korea), thin films of Cr, Au, and SiO_2_ were deposited on the cleaned glass slide at a rate of 0.5 Å/s at a pressure of 5 × 10^−6^ Torr (Figure 1b). Commercial Cr (99.9%), Au (99.9%), and SiO_2_ (99.99%) granules of sizes 1–3 mm used as the source materials for EB-PVD were purchased from Taewon Scientific Co., Ltd (Seoul, Republic of Korea). The samples were prepared with Au layer thickness ranging from 30 to 200 nm and Cr and SiO_2_ layer thickness of 4 and 10 nm, respectively. The sample stage was rotated at 7 rpm during deposition to achieve uniform layer thickness. The thickness of the deposited layers was monitored during deposition using a quartz crystal microbalance and then measured using an atomic force microscope (AFM) (XE7, Park Systems, Suwon, Republic of Korea). Then, a QD layer with a thickness of approximately 17 nm was spin-coated onto the prepared thin-film samples at 5000 rpm for 30 s, as shown in Figure 1c. The QD solution had a concentration of 25 mg/mL. In addition, a reference sample was prepared without the Au layer. After spin coating, the QD layer was irradiated with an e-beam. Both imaging and EBL were performed with a field emission electron gun (SEM MIRA3, TESCAN, Brno, Czech Republic) (Figure 1d). For EBL, the accelerating voltage was 30 kV, the working distance was 3 mm, and the scan rate was 0.32 µs/px. The beam current was set to 6 pA. Immediately after exposure, the samples were immersed in toluene for 10 s (Figure 1e). To allow a fair and consistent comparison between different e-beam dosages, we intentionally kept a constant duration of toluene immersion. Increasing the toluene immersion duration for each dosage would have resulted in a significant increase in the duration and complexity of the experiment, as this would have required the study of multiple dosage samples with different immersion durations. The QDs were used as negative electron resists because the ligand of the QDs, oleic acid, can form cross-links and strong bonds when exposed to high energy density. A detailed description of the experimental procedure can be found in the electronic Supplementary Information (ESI), see Figures S1 and S2.

## 3. Results and Discussion

### 3.1. Electron-Beam Exposure Dose Test

**Figure 2:**
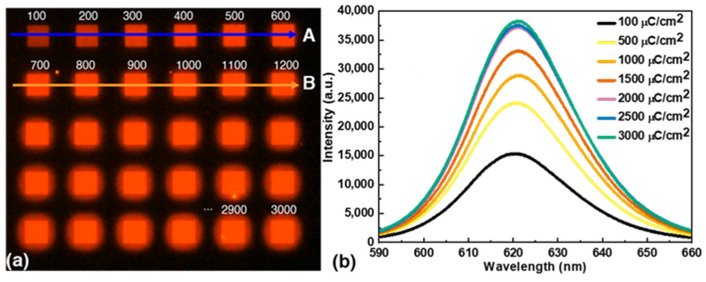
(**a**) Fluorescence micrographs of patterned QDs at increasing e-beam doses from 100 to 3000 μC/cm^2^. Figure S3 (ESI) shows the line profiles of the first two rows (A and B) of the patterns confirming the effect of e-beam dose on micropattern properties, such as changes in micropattern height profiles, surface topology, and fidelity. (**b**) QD fluorescence spectra at varying e-beam exposure dose.

During the pattern formation process, irradiation with e-beams can lead to a decrease in QD fluorescence [21]. Therefore, we investigated the effects of e-beam dose on QD fluorescence to find the optimal dose for pattern formation that does not affect nanopattern quality or fluorescence. QD patterns (8 × 8 µm^2^) were generated with e-beam doses ranging from 100 to 3000 µC/cm^2^ (Figure 2a). A fluorescence microscope (LS40, LEAM Solution, Siheung, Republic of Korea) was used to obtain images at a magnification of 400× with a light source of 425 nm wavelength and a 625 nm emission filter. The e-beam dose was gradually increased in steps of 100 µC/cm^2^ as shown in Figure 2a from the left corner. Figure 2b shows the relationship between the fluorescence spectra of the QD patterns and the exposure doses. The fluorescence spectra were measured using a confocal spectrofluorometer (ACRON, UniNanoTech, Yongin, Republic of Korea) with an excitation laser of wavelength 375 nm.

The QD pattern generated with a dose of 100 µC/cm^2^ exhibited lower fluorescence intensity and the fluorescence intensity increased with increasing e-beam dose, reaching saturation at 2000 µC/cm^2^. The observed increase in fluorescence intensity of the QD nanopatterns at higher e-beam doses can be attributed to the increased cross-linking of QDs due to the stronger e-beam irradiation. This phenomenon has already been observed in previous studies with organic layers [22,23]. The formation of additional cross-links between QDs leads to a thicker pattern, which in turn increases the fluorescence intensity of the QD nanopattern. However, it is crucial to carefully select the optimal e-beam dose to avoid overexposure and to obtain high pattern fidelity.

The fluorescence images of the micropatterns clearly showed the effects of the different e-beam doses on pattern fidelity with significant differences between the actual dimensions and the original input dimensions (Figure 2a). At doses below 500 µC/cm^2^, the fluorescence intensity was lower than the maximum achievable value, indicating incomplete pattern formation. Doses greater than 1000 µC/cm^2^, on the other hand, resulted in decreased pattern fidelity, with micropatterns deviating significantly from target dimensions. However, at a dose of 500 µC/cm^2^, the micropatterns exhibited high fidelity, closely resembling the target dimensions and reaching approximately 70% of the maximum fluorescence intensity. With this dose, the right balance was found, allowing accurate replication of the pattern with sufficient fluorescence intensity.

The line profiles derived from the fluorescence images also confirmed the effect of e-beam dose on micropattern properties, such as changes in micropattern height profiles, surface topology, and fidelity. Figure S3 (ESI) shows the line profiles of the first two rows (A and B) of the patterns shown in Figure 2a. The 500 µC/cm^2^ dose produced a micropattern with a full width at half maximum (FWHM) of 7.93 µm, which matched the target size of 8 µm. In contrast, the 700 µC/cm^2^ dose resulted in a slightly larger FWHM along line B, indicating a deviation from the target size. In addition, increasing the dose to 1200 µC/cm^2^ resulted in a wider pattern with a width of 8.67 µm. These results indicate that higher e-beam doses can affect the dimensional accuracy of the micropatterns, potentially leading to deviations from desired specifications. Importantly, the micropatterns fabricated with a dose of 500 µC/cm^2^ had sharp edges and minimal background fluorescence outside the patterned area. This indicates that the e-beam irradiation was confined precisely to the desired area, resulting in well-defined micropatterns with minimal interference from scattered radiation. Consequently, a dose of 500 µC/cm^2^ was chosen as the optimal dose for QD EBL in this study, as it can achieve a balance between fluorescence intensity and micropatterns fidelity.

### 3.2. Enhanced Fluorescence of QD Micropatterns

**Figure 3:**
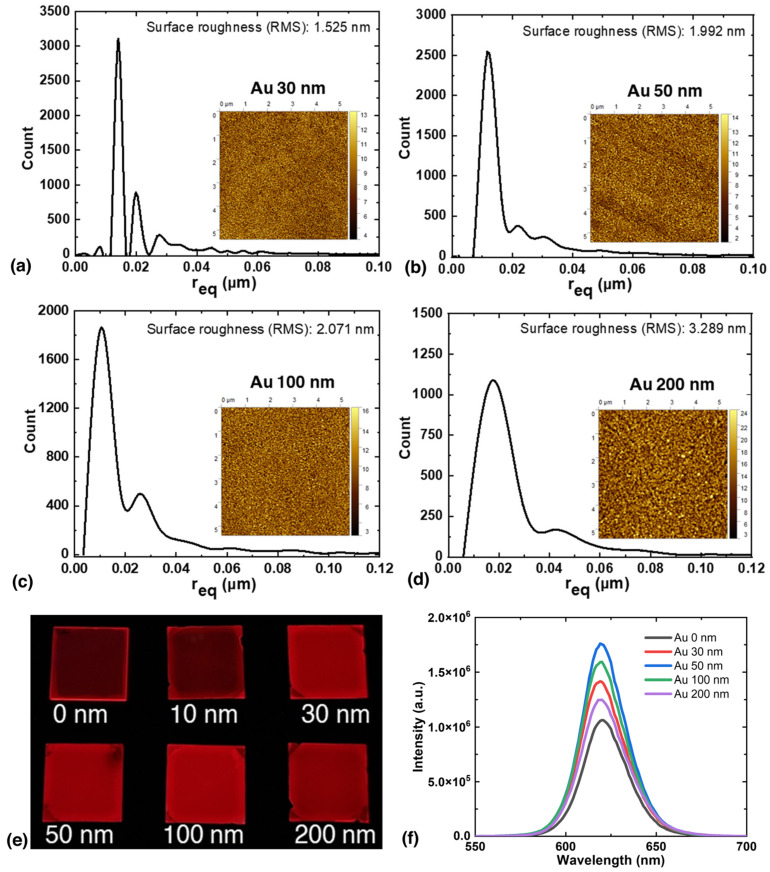
Grain distribution of Au layers. Equivalent disk radius (r_eq_) of the surface features on (**a**) 30 nm, (**b**) 50 nm, (**c**) 100 nm, (**d**) 200 nm Au layers. (**e**) I Fluorescence images of spin-coated QDs on Au layers of different thicknesses. (**f**) Fluorescence spectra of spin-coated QDs on Au layers of different thicknesses. For the fluorescence measurements, a 10 nm SiO_2_ spacer layer was deposited over the Au layer for all samples.

Earlier studies have shown that SP on Au surfaces can enhance the fluorescence signal [24,25]. Renier et al. showed that a simple architecture consisting of a glass substrate, an Au layer, and an SiO_2_ layer can enhance the fluorescence signal emitted from a fluorophore on the surface without the need for nanostructuring the surface [26,27]. Therefore, in this study, we deposited Au and SiO_2_ layers as substrates for QD nanolithography to further enhance QD fluorescence. However, the fluorescence intensity is affected by the distance between the QD and the Au layer as well as the properties of the Au layer [28,29,30]. Moreover, the change in fluorescence observed near a metal surface is primarily due to variations in the quantum yield and lifetime of fluorescence, which are sensitive to the distance between the metal layer and the QDs [31]. Previous studies have shown that the optimal distance for maximum enhancement between QDs and metal is typically in the range of 8–12 nm [32,33,34]. To achieve this optimal distance, we deposited a 10 nm SiO_2_ spacer layer over the Au layer before depositing the QD layer. In agreement with previous results, we observed that the fluorescence intensity increased only when the SiO_2_ spacer layer was present, as shown in Figure S4 (ESI). This can be attributed to the fact that energy transfer to the metal without the SiO_2_ layer causes quenching, which leads to an increase in nonradiative recombination [35,36]. In addition, the fluorescence enhancement can be significantly affected by the thickness and roughness of the Au layer [37,38]. In our study, we varied the thickness of the Au layer from 30 to 200 nm and observed corresponding changes in roughness, ranging from 1.525 nm for the 30 nm Au layer to 3.289 nm for the 200 nm Au layer (Figure 3a–d). We did not focus on the roughness but investigated the effect of varying the Au layer thickness, since we could control the layer thickness during the deposition process. In the following section, we discuss the effects of Au layer thickness on the enhancement of QD fluorescence.

Figure 3e shows photographs of QDs spin-coated on Au layers of different thicknesses, taken in a darkroom with a 365 nm LED light source (M365L3, Thorlabs Inc., Newton, NJ, USA). For fluorescence measurements, a 10 nm SiO_2_ spacer layer was deposited over the Au layer for all samples. In our study, we specifically focused on investigating the effect of Au layer thickness on fluorescence enhancement, as previous research has shown that Au layer roughness (thickness) can affect the fluorescence intensity of the QDs. By keeping the SiO_2_ thickness constant, we aimed to isolate the effect of SiO_2_ thickness on fluorescence enhancement and investigate the influence of only Au layer thickness in a controlled manner. Including roughness data of the SiO_2_ layer at different Au thicknesses might introduce additional variables that could complicate the interpretation of our results. When observed with the naked eye, the fluorescence intensity without Au (0 nm) was relatively low compared to that of Au layers with a thickness of 30–200 nm. The fluorescence spectra of the spin-coated QDs layers are shown in Figure 3f. We observed an increase in fluorescence intensity with increasing thickness of the Au layer, and at 50 nm, the fluorescence intensity was approximately 1.75 times higher than without the Au layer. However, the fluorescence intensity did not increase with increasing Au layer thickness beyond 50 nm, but decreased, and the QDs on the 200 nm Au layer had lower fluorescence intensity than those on the 30 nm layer. The shift in absorbance with increasing Au layer thickness affected the excitation conditions for SPs (Figure S5, ESI). In agreement with previous studies, we found that the 50 nm Au layer thickness gave the highest fluorescence enhancement [39,40].

**Figure 4:**
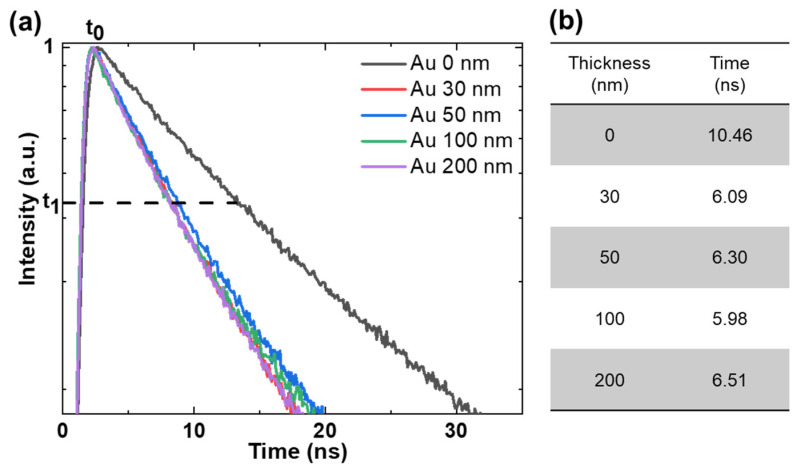
(**a**) Fluorescence decay curve of QDs with and without Au layers of different thickness. (**b**) Fluorescence lifetime of QDs with and without Au layers of different thickness. For all samples, a 10 nm SiO_2_ spacer layer was deposited over the Au layer.

To investigate the influence of QD-SP interactions on the emission rate, further experiments were performed using a time-resolved spectrofluorometer (Fluorolog-QM, HORIBA Scientific, Piscataway, NJ, USA). Figure 4 shows the representative observations of the fluorescence lifetime (1/e) of the glass and Au layers at an excitation wavelength of 375 nm.

The influence of Au layer thickness on the photoluminescence (PL) of QDs has been extensively studied in previous studies [27,41]. The relationship between Au layer thickness and QD PL is complex and depends on several factors. However, some studies suggest that thin Au layers can enhance the interaction between the QDs and the Au layer, leading to an increase in PL intensity. Conversely, thicker Au films can suppress QD emission, leading to a decrease in PL intensity. In these studies, an exponential decrease in the SPR-induced local field was also observed with increasing Au film thickness, with defects identified as the main factors affecting the coupling between SP resonane and the QD band structure. In our study, the lifetime of QDs on Au layers was shorter than that of QDs on glass substrates, indicating exciton SP coupling [42]. Although it may seem counterintuitive that the 0 nm sample with the lowest PL intensity has the longest lifetime, this phenomenon can be explained by the presence of SP–exciton coupling and the contribution of the propagating SP modes. The exciton–SP coupling arises from the interaction between the QDs and the propagating SP modes supported by the Au layer. These SP modes increase the recombination rate of the QDs, leading to an increase in the emission intensity observed in the PL measurements for the Au-coated samples. The shorter lifetime of QDs on Au layers compared to glass substrates, in combination with the enhanced recombination by SP modes, contributes to the higher PL intensity observed in the Au-coated samples. In addition, there is a strong correlation between the measured enhancement factor of 1.7 for fluorescence efficiency and the reduction factor of 1.7 for lifetime observed for the 50 nm Au layer. Under ideal conditions, the lifetime reduction factor is expected to be equal to the fluorescence enhancement factor [43]. However, for Au layers of different thicknesses, tIe interaction between QDs and SPs may introduce an additional nonradiative recombination channel that reduces the extent of this enhancement [41]. The non-radiative recombination channel formed by the interaction between QDs and SPs at different thicknesses of the Au film can be affected by factors such as material properties, QD-SP spacing, and fabrication techniques. Consequently, extensive experimental studies and characterizations are critical for optimizing the coupling between QDs and SPs to achieve maximum radiative enhancement while minimizing non-radiative losses. However, it is important to note that in this study, our main focus was on the direct fabrication of QD nanopatterns using e-beam lithography, and such experimental and characterization analysis are beyond the scope of the present study. Therefore, further research is needed to accurately elucidate the alternative nonradiative recombination pathways involved in the interaction between QDs and SPs.

### 3.3. Direct Patterning of QDs Using E-Beam Lithography

**Figure 5:**
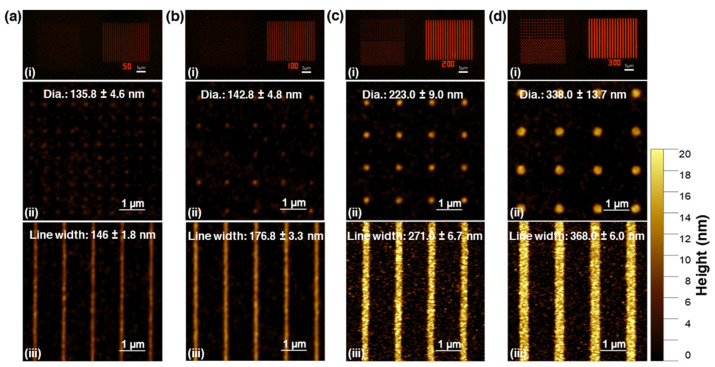
Fluorescence images (**a**–**d**(**i**)) and AFM images of dot (**a**–**d**(**ii**)) and line patterns (**a**–**d**(**iii**)) of QDs fabricated by e-beam lithography.

In this section, we demonstrate the practicality of direct QD patterning with EBL. We created line and dot patterns of varying widths and diameters using AutoCAD software. The QD layer was then exposed to an e-beam and developed in toluene. Figure 5 shows the fluorescence images of QD nanopatterns with different sizes. Comparing Figure 5a–d we observe that the fluorescence images appear brighter for the patterns with larger width. For example, the fluorescence images in Figure 5c,d appear brighter than those in Figure 5a,b, which have smaller widths. To confirm this observation, AFM was used to measure the height of the line and dot patterns. The line patterns in Figure 5a,d had heights of 9 nm and 17 nm, respectively, while the dot patterns in Figure 5a,d had heights of 5 nm and 15 nm, respectively. Despite the identical e-beam dose of 500 µC/cm^2^, the height of the patterns varied. This discrepancy can be attributed to the limited resolution of the instrument, which resulted in line patterns with a minimum size of 135 nm and dot patterns with a minimum size of 145 nm—both more than twice the size specified in the design files.

To assess the feasibility of e-beam patterning in the presence of the SiO_2_ layer on the Au layers, we compared the fluorescence intensities of the QD patterns with and without the SiO_2_ layer. Although the top layer was 10 nm insulating SiO_2_, no effect of e-beam charging was observed. Typically, the penetration depth of an e-beam is approximately 1 µm, depending on the energy of the beam [44,45]. Due to the conductive Au layer under the thin SiO_2_ layer, energy transfer took place while the charging effect was attenuated.

**Figure 6:**
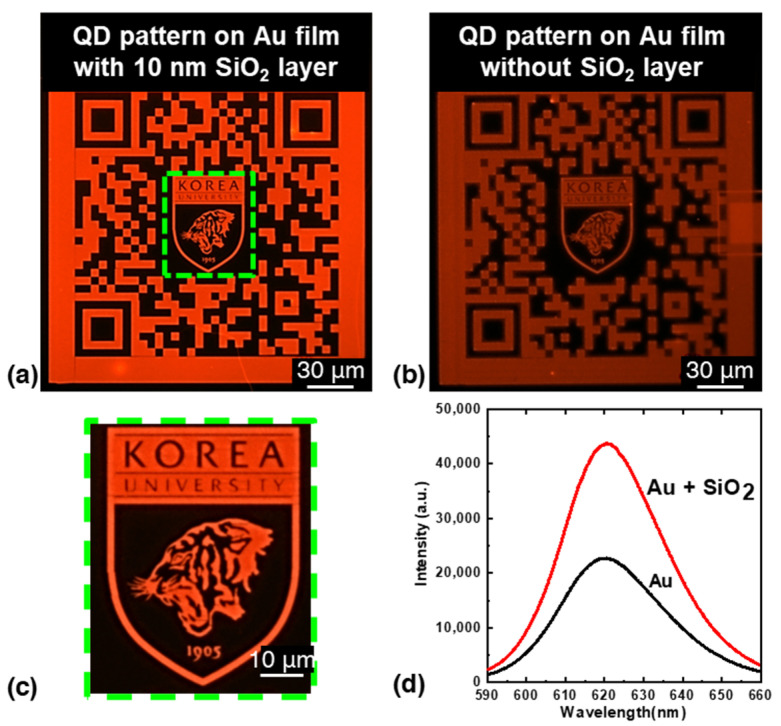
Fluorescence image of the QR code of Nano Bio Photonics Lab, Korea University, patterned at 500 µC/cm^2^ by direct e-beam lithography (**a**) with SiO_2_ layer; (**b**) without SiO_2_ layer; (**c**) magnified image of (**a**); (**d**) fluorescence spectra of the QR pattern of Nano Bio Photonics Lab, Korea University with and without SiO_2_ layer.

In addition, we investigated the direct patterning of intricate patterns, such as quick response codes (QR), with QDs. Figure 6 shows the QR code for the website of the Nano-Bio-Photonics Laboratory at Korea University. Figure 6a,b show the patterned QD QR code deposited on a 50 nm Au layer with and without SiO_2_ layer, respectively.

A comparison of the fluorescence microscopy images with and without the SiO_2_ layer at the same exposure time of 6 ms shows a significantly higher fluorescence intensity for the pattern with the SiO_2_ layer. The presence of SiO_2_ as an interlayer between the QD and the Au layer helps to minimize quenching effects and increase fluorescence intensity by reducing non-radiative decay processes. Figure 6c shows a magnified view of the green dotted box in Figure 6a, where the fine whiskers of the Korea University tiger logo, including the minimum line width of 160 nm, are clearly visible. After development, the fluorescence spectra of the QD patterns on the 50 nm Au layer with and without SiO_2_ layer are shown in Figure 6d. The QD pattern with SiO_2_ exhibited approximately twice the fluorescence intensity of the pattern without SiO_2_. In addition, the absence of background fluorescence in Figure 6a indicates that all unexposed QDs were successfully removed from the substrate.

## 4. Conclusions

In summary, we successfully demonstrated the direct patterning of commercial QDs with EBL. We investigated the effects of e-beam dose on the fluorescence intensity and fidelity of QD nanopatterns. We found that increasing the e-beam dose resulted in higher fluorescence intensity and improved pattern visibility, up to a saturation point beyond which pattern accuracy and fidelity decreased. By carefully selecting an optimal dose of 500 µC/cm^2^, we achieved micropatterns with high accuracy and approximately 70% of the maximum fluorescence intensity. The presence of Au and SiO_2_ layers as substrates further enhanced the fluorescence of the QD patterns. We observed that the thickness and roughness of the Au layer played a crucial role in fluorescence enhancement, with a thickness of 50 nm exhibiting the highest enhancement factor. In addition, we successfully demonstrated the direct patterning of complex patterns, such as QR codes, with QDs. The combination of Au and SiO_2_ layers proved effective in achieving increased fluorescence intensity and pattern clarity.

In conclusion, our results highlight the feasibility and effectiveness of direct QD nanopatterning with EBL. By optimizing the e-beam dose and interlayer materials, we were able to generate patterns with high fidelity and increased fluorescence intensity. This research paves the way for the development of advanced nanofabrication techniques for applications in nanophotonics, biosensing, and optoelectronic devices.

## Data Availability

The data presented in this study are available on request from the corresponding author.

## Supplementary Materials

The following supporting information can be downloaded at: https://www.mdpi.com/article/10.3390/nano13142111/s1, Figure S1: Layout design of QR, dot, and line patterns for e-beam lithography; Figure S2: Formation of cross-links by recombination of radicals in adjacent polymer chains; Figure S3: (a) Fluorescence micrographs of patterned QDs at increasing e-beam doses from 100 to 3000 μC/cm^2^. Fluorescence intensity line profile of patterned QDs from (b) row A and (c) row B in Figure (a); Figure S4: The fluorescence image of spin-coated QD on (a) glass, Au layer, and Au layer with 10 nm SiO2 spacer layer. (b) Fluorescence spectra of spin-coated QDs on Au layer with and without SiO2 spacer layer. The QDs on the Au layer with SiO2 layer show an increased fluorescence efficiency, which is about twice as high as that of the QDs on the glass with or without Au layer; Figure S5: (a) Experimental absorption spectra and (**b**) absorption spectra determined with FDTD for different Au thicknesses. Refs. [11,46,47,48,49,50,51,52] are cited in the Supplementary Materials.
