# Supplementary material for: Colloidal Quantum Dot Nanolithography: Direct Patterning via Electron Beam Lithography
Source: Nanomaterials (Basel). 2023 Jul 20;13(14):2111. doi: 10.3390/nano13142111 (PMC10384559; doi:10.3390/nano13142111)
Supplement: Supplementary file 1 [file nanomaterials-13-02111-s001.zip › nanomaterials-2506538-supplementary.pdf]

nanomaterials

Supplemental Information

Colloidal Quantum Dot Nanolithography: Direct Patterning via Electron Beam Lithography

Taewoo Ko,‡ Samir Kumar,‡ Sanghoon Shin, Dongmin Seo,* and Sungkyu Seo*

1. E-beam lithography process parameters

To ensure accurate e-beam exposure without astigmatism-related distortion, a stigmator was calibrated with eight electromagnets. In addition, the focus and spot size of the e-beam were optimized with a wobbler to avoid beam oscillations. After irradiation with the e-beam, the quantum dot patterns (QDs) were developed in toluene. Subsequently, the QDs were immersed in toluene for 15 seconds with gentle agitation and then rinsed with deionized water and dried with a nitrogen gun.

Unlike photolithography, electron beam lithography (EBL) does not require a separate photomask. Instead, the electron beam is used directly. Figure S1 shows the layout design of the QD patterns using ELPHY Quantum, a computer-aided design application developed by Raith GmbH (CAD). Figure S1a shows the pattern file in the form of a QR code design. The size of the pattern is (200 × 200) μm, and the width of the Tiger's Whisker pattern is 160 μm. Figures S1b and S1c also show dot and line patterns created with a diameter and line width of 100 μm, respectively.

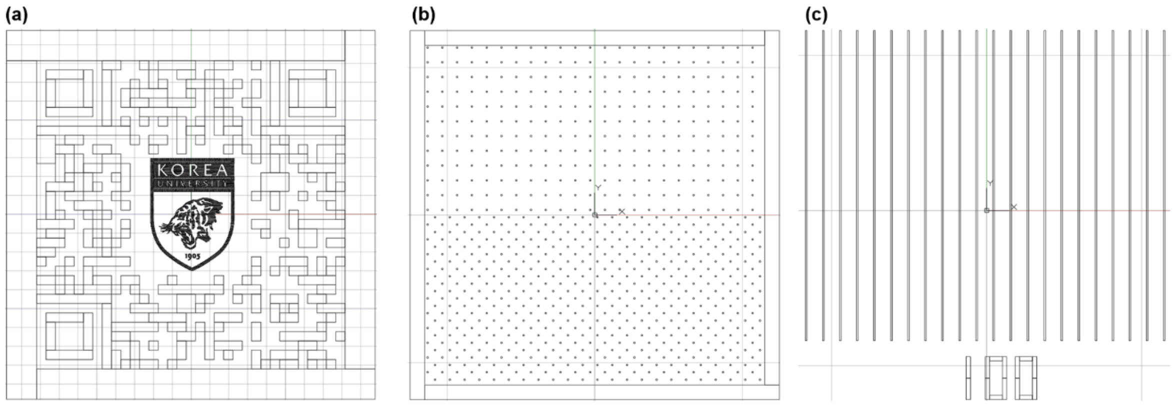

Figure S1. Layout design of quick response (QR), dot, and line patterns for electron beam lithography.

2. Cross-linking between quantum dots

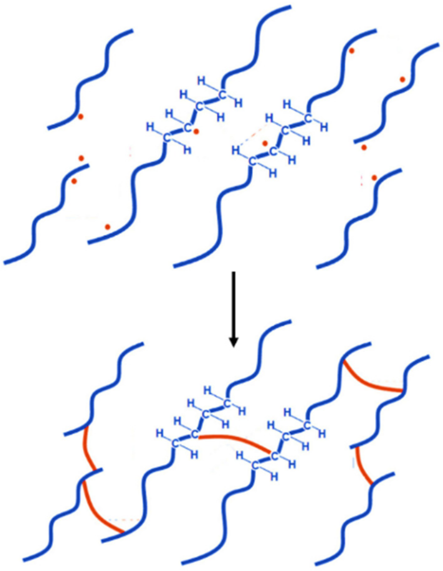

Figure S2. Formation of cross-links by recombination of radicals in adjacent polymer chains [1].

Oleic acid is a widely used ligand for the surface modification of QDs. The term crosslinking refers to the chemical process by which covalent bonds are formed between adjacent polymer chains, leading to the formation of larger QD clusters or aggregates. This process is often triggered by free radicals, which are highly reactive species that can form new chemical bonds with other molecules or radicals. In QD systems, oleic acid can serve as a crosslinking agent due to its carboxylic acid functional group. This functional group can react with other functional groups on the QD surface or with other oleic acid molecules. Related polyethylene structures may also explain the cross-linking after oleic acid is irradiated with high energy. Irradiation of polyethylene generates free hydrogen and alkyl radicals with the configuration-CH_2-•CH-CH_2- [1]. These radicals have an unshared electron that was previously attached to the hydrogen atom. When two such free radicals form on adjacent or connected chains, their recombination often results in crosslinking. Common chemical bonds such as C-C and C-H have bond strengths of 3.6 eV and 3.3 eV, respectively. Even electrons with energy far below this bond strength can cause chemical changes through dissociative electron attachment [2,3]. Ionizing e-beam breaks bonds in polymer molecules, and when these bonds are repaired, crosslinking occurs. Ligands of QDs and monomers such as oleic acid are cross-linked by high energy density electromagnetic radiation and EUV radiation [4]. It has been observed that electron irradiation has an effect similar to that of high-energy photons. Therefore, the electron irradiated regions remain firmly bonded to the substrate after development, suggesting that monomers such as oleic acid can act as electron resists when incorporated as ligands in the lithography process using an electron beam.

3. e-beam exposure dose test

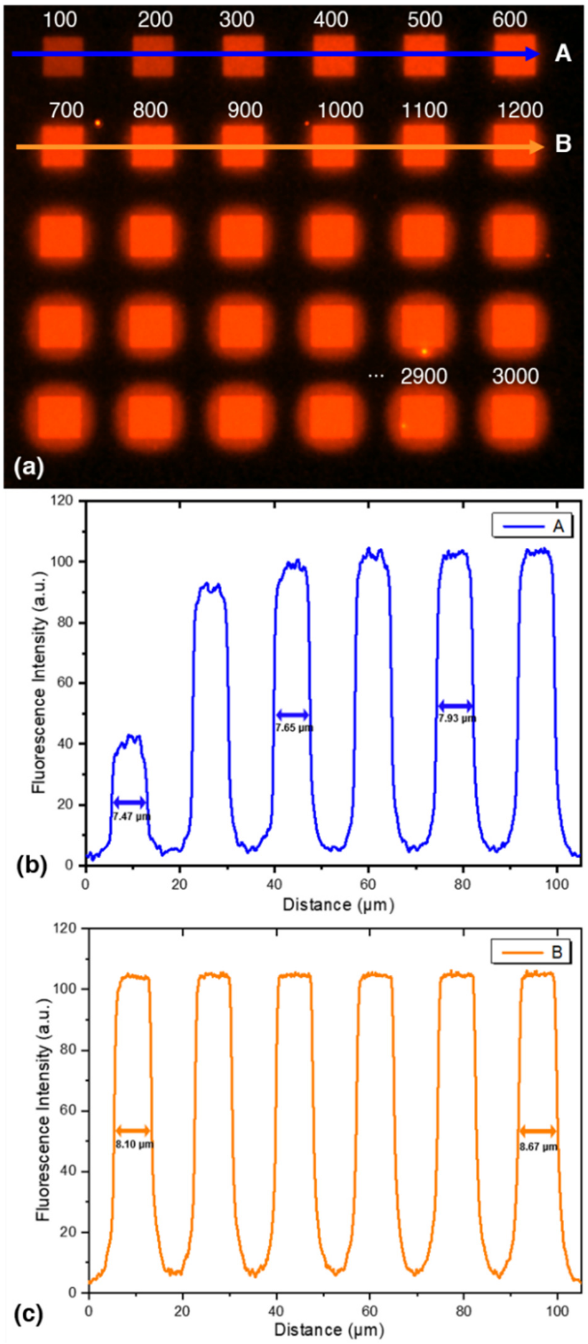

Figure S3. (a) Fluorescence micrographs depicting patterned quantum dots (QDs) at increasing electron beam doses ranging from 100 to 3000 μC/cm^2; (b) Line profile of fluorescence intensity showing the variation in signal along row A of the patterned QDs shown in Figure (a); (c) Line profile of fluorescence intensity demonstrating the change in signal along row B of the patterned QDs displayed in Figure (a).

4. Enhanced fluorescence from spin-coated QD

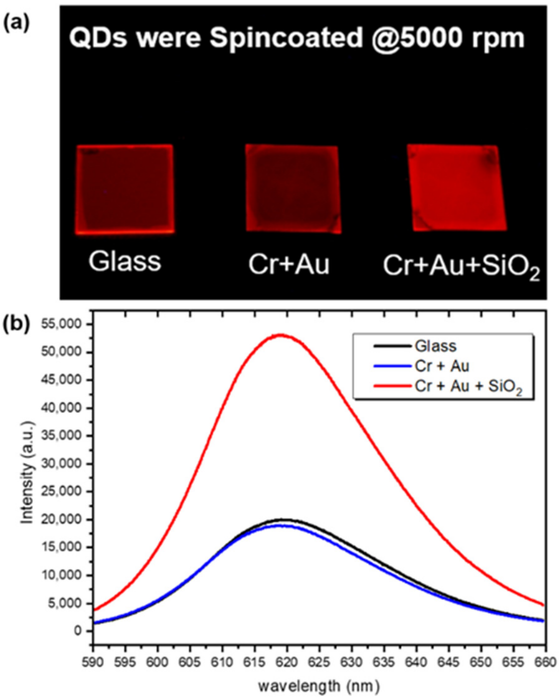

Figure S4. (a) Fluorescence image of spin-coated QDs on different substrates: glass, Au layer, and Au layer with a 10 µm SiO_2 spacer layer. (b) Fluorescence spectra of spin-coated QDs on the Au layer with and without the SiO_2 spacer layer. The QDs on the Au film with the SiO_2 layer exhibit significantly enhanced fluorescence efficiency, approximately twice as high as that of the QDs on glass substrates, regardless of the presence or absence of an Au layer.

5. Optical properties of Au thin film

The optical properties of the multilayer film composed of Cr, Au, and SiO2 were investigated by experimental measurements and finite-difference time-domain (FDTD) simulations to determine its absorption. Shimadzu UV-Vis spectrometer (UV-2600) was used for experimental absorbance measurement in reflectance mode with a resolution of 1 µm, covering the wavelength range from 350 nm to 700 nm. Absorbance was measured for different Au thicknesses (30 µm, 50 µm, 100 µm, and 200 µm) and for spin-coated QD film on glass.

For the FDTD simulations, Lumerical's automatic non-uniform crosslinking algorithm was used with a minimum grating step of 10 µm. The Lumerical integrated material models for gold and silica were used for the simulations [5–7]. To ensure accurate results and convergence, automatic non-uniform meshing was used with the finest meshes (minimum mesh size of 0.25 µm) and auto-cut-off at 1×10^{-5}. The simulation time was set at 1000 µs, and convergence was usually achieved before the simulation time expired. The light source covered a wavelength range from 350 nm to 700 nm.

The perfectly matched layer boundary condition was applied outside the simulated region in both x and y directions, and the incident radiation propagated in the negative z direction (toward the bottom of the nanorods), similar to the actual experiment.

The experimental results showed that the absorption of the Au film peaked at 500 nm and exhibited a slight blue shift as the film thickness increased [8]. The simulated results also showed an absorption peak at 500 nm and a blue shift of the peak with increasing Au thickness, which is consistent with the experimental observations. However, a broad peak observed experimentally at 25 nm Au thickness was not clearly detected in the FDTD simulation. This discrepancy could be due to the fact that the simulation does not take into account the influence of surface roughness.

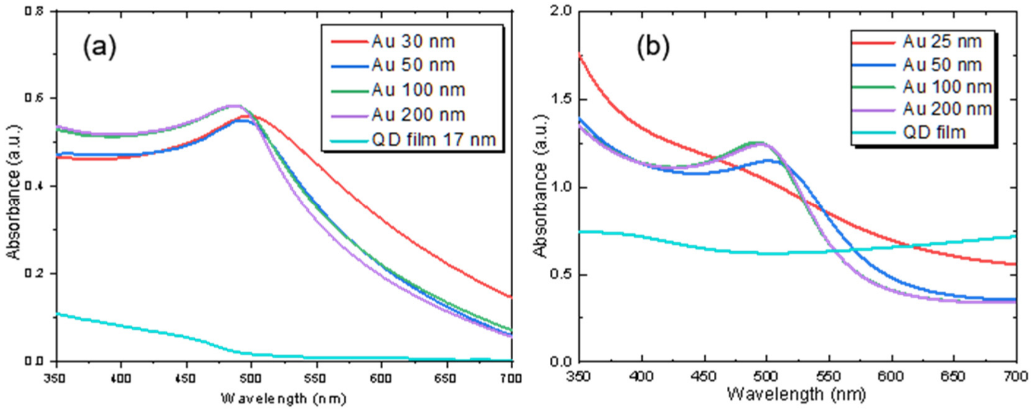

Figure S5. (a) Experimental absorption spectra and (b) absorption spectra determined using Finite-Difference Time-Domain (FDTD) simulations for various thicknesses of Au.

References

1. Patterson, R.; Kandelbauer, A.; Müller, U.; Lammer, H. Crosslinked Thermoplastics. In *Handbook of Thermoset Plastics*; Elsevier, 2014; pp. 697–737.
2. Kozawa, T.; Tagawa, S. Radiation Chemistry in Chemically Amplified Resists. *Jpn. J. Appl. Phys.* **2010**, *49*, 030001, doi:10.1143/jjap.49.030001.
3. Narasimhan, A.; Wisehart, L.; Grzeskowiak, S.; Ocola, L.E.; Denbeaux, G.; Brainard, R.L. What We Don't Know about EUV Exposure Mechanisms. *J. Photopolym. Sci. Technol.* **2017**, *30*, 113–120, doi:10.2494/photopolymer.30.113.
4. Dieleman, C.D.; Ding, W.; Wu, L.; Thakur, N.; Bespalov, I.; Daiber, B.; Ekinci, Y.; Castellanos, S.; Ehrler, B. Universal direct patterning of colloidal quantum dots by (extreme) ultraviolet and electron beam lithography. *Nanoscale* **2020**, *12*, 11306–11316, doi:10.1039/d0nr01077d.
5. Rajput, A.; Kumar, S.; Singh, J.P. Vertically standing nanoporous Al--Ag zig-zag silver nanorod arrays for highly active SERS substrates. *Analyst* **2017**, *142*, 3959–3966, doi:10.1039/C7AN00851A.
6. FDTD Available online: http://www.lumerical.com/tcad-products/fdtd/. (accessed on 27 February 2023).
7. Kumar, S.; Doi, Y.; Namura, K.; Suzuki, M. Plasmonic Nanoslit Arrays Fabricated by Serial Bideposition : Optical and Surface-Enhanced Raman Scattering Study. *ACS Applied Bio Materials* **2020**, *3*, 3226–3235, doi:10.1021/acsabm.0c00215.
8. Axelevitch, A.; Apter, B. In-Situ Investigation of Optical Transmittance in Metal Thin Films. *Thin Solid Films* **2015**, *591*, 261–266, doi:10.1016/j.tsf.2015.01.046.
